# Suitability Evaluation of Multipoint Simultaneous CO_2_ Sampling Wireless Sensors for Livestock Buildings

**DOI:** 10.3390/s140610479

**Published:** 2014-06-13

**Authors:** Salvador Calvet, José Carlos Campelo, Fernando Estellés, Angel Perles, Ricardo Mercado, Juan José Serrano

**Affiliations:** 1 Institute for the Applications of Advanced Information and Communications Technologies (ITACA), Universitat Politècnica de Valencia, Camino de Vera, 46022 Valencia, Spain; E-Mails: jcampelo@itaca.upv.es (J.C.C.); rmercado@itaca.upv.es (R.M.); jserrano@itaca.upv.es (J.J.S.); 2 Institute of Animal Science and Technology, Universitat Politècnica de València, Camino de Vera, 46022 Valencia, Spain; E-Mails: salcalsa@upv.es (S.C.); feresbar@upv.es (F.E.)

**Keywords:** carbon dioxide, livestock, ventilation, wireless sensor network

## Abstract

The environment in livestock buildings must be controlled to ensure the health and welfare of both workers and animals, as well as to restrict the emission of pollutants to the atmosphere. Among the pollutants generated inside these premises, carbon dioxide (CO_2_) is of great interest in terms of animal welfare and ventilation control. The use of inexpensive sensors means that complete systems can be designed with a number of sensors located around the building. This paper describes a study of the suitability of multipoint simultaneous CO_2_ sensors operating in a wireless sensor network, which was found to operate satisfactorily under laboratory conditions and was found to be the best alternative for these applications. The sensors showed a highly linear response to CO_2_ concentrations, ranging from 500 to 5000 ppm. However, individual sensor response was found to differ, which made it necessary to calibrate each one separately. Sensor precision ranged between 80 and 110 ppm CO_2_, and sensor response to register a 95% change in concentration was estimated at around 5 min. These features mean this type of sensor network can be used to monitor animal welfare and also for environmental control in poorly ventilated livestock premises. According to the tests conducted in this study, a temporal drift may occur and therefore a regular calibration of sensors would be needed.

## 1. Introduction

Intensive livestock rearing is associated with the emission of large amounts of atmospheric pollutants, which impair animal health and welfare inside the buildings and have a serious environmental impact. The accumulation of ammonia (NH_3_) inside livestock houses affects animal health and welfare [1,2], and the emission of this gas is associated with the acidification of soils and the eutrophication of ecosystems [3]. Greenhouse gases such as methane (CH_4_) and nitrous oxide (N_2_O), which are generated both by the animals themselves and their manure, contribute to climate change [4]. Due to the accumulation of these effects, many regulations have been issued in recent years (e.g., the Kyoto Protocol, European Ceilings Directive), which directly or indirectly affect the design and operation of livestock buildings.

Carbon dioxide (CO_2_) is produced in livestock farms mainly as a result of animal respiration and the aerobic decomposition of their manure. Although CO_2_ is a greenhouse gas, animal-related CO_2_ emissions are not considered a net addition to the atmosphere, since this gas participates in a short-term closed cycle [5]. However, CO_2_ has been widely used as a tracer gas to quantify ventilation in livestock buildings [6] and exposure to high concentrations of this gas has also been associated with impaired health of both humans and animals [7].

Ventilation of livestock housing provides fresh air to the animals and removes any potential airborne pollutants released inside the building. Measuring ventilation rates in livestock buildings is therefore essential for proper building design and operational control. Furthermore, ventilation should be quantified and the air should be monitored with a view to determining gas concentrations in order to estimate the emission of other atmospheric pollutants by means of mass balances. Currently, most pollutants can be measured with acceptable precision using a wide variety of measurement techniques [8], but quantifying airflow rates may be more complicated [9].

Measuring ventilation rates in commercial livestock buildings is complex and time-consuming, and is particularly challenging in naturally ventilated livestock buildings, where changing outdoor conditions determine irregular and continuously varying ventilation patterns. Naturally ventilated buildings are commonly used to house certain animal species such as dairy cattle and are widely used for other species under mild or warm conditions. They are also seen as a good solution in developing countries and areas with restricted access to electricity. However, the measurement and control of a ventilation system remains an elusive target for farmers and researchers [10]. As discussed by [9], CO_2_ can be used as a tracer to determine farm ventilation and pollutant emissions, but more development is needed regarding CO_2_ production inside the farm and also in terms of CO_2_ measurement technology.

Farmers are increasingly required to improve the control of ventilation in their buildings. Welfare regulations also include a ventilation system which is able to maintain indoor gas concentrations below certain thresholds (e.g., 20 ppm NH_3_ and 3000 ppm CO_2_ for broilers, according to European Council Directive 2007/43/EC [11]. It is also now widely accepted that maintaining appropriate air quality in animal housing is effective, not only to ensure acceptable welfare status, but it also has a positive influence on animal performance.

Measuring CO_2_ concentrations in livestock buildings is therefore of great interest to farmers. Firstly, this information could be included in the farm climate control system to control ventilation as required. Sly, the accurate determination of ventilation rates by means of CO_2_ balances would allow alternative housing systems to be characterized in terms of pollutant emissions. In addition, such a system would provide information on animal welfare related to exposure to gaseous pollutants.

CO_2_ balance has been proposed as alternative method of estimating ventilation rates. The CO_2_ naturally emitted in livestock houses fulfills the properties of a tracer gas established by [12]: low and stable background concentrations, low risk in case of exposure, easy to handle and measure. The CO_2_ balance methodology has been widely described [6,13] and applied to a number of mechanically ventilated livestock houses. To apply this method two inputs must be characterized accurately. The first requires that the CO_2_ produced by the animals and their manure be well characterized; this production can be estimated according to [6] and is proportional to the metabolic activity of the animals, which depends on the type of animal, its weight and its productivity. The s input is an accurate measure of the difference in CO_2_ concentration between the exhaust and incoming air. This requires a high time and space resolution in concentration measurements.

The air inside livestock houses is characterized by relatively low CO_2_ concentrations, which are mostly under 5000 ppm [14–17]. In some cases (naturally ventilated, open buildings) indoor CO_2_ concentrations are lower than 500 ppm, which involves a difference of less than 200 ppm with outdoor air. In most mechanically ventilated buildings the incoming air mixes perfectly with the air already present and CO_2_ concentration gradients are stable and well characterized. However, in naturally ventilated buildings (particularly those of open structure), indoor and outdoor concentrations are similar, perfect mixing is not achieved and permanent changes in CO_2_ gradients are common as a consequence of changes in airflow patterns. CO_2_ concentration measurements in livestock buildings must meet the following requirements:

- They should be sufficiently precise for the purpose of the study. For assessing animal welfare, a precision of 100 ppm may be enough, considering that a threshold of 3000 to 5000 ppm is normally established. For ventilation, the precision should be enough to properly characterize the difference between the indoor and outdoor environments. In closed, naturally ventilated buildings, a difference of 150 ppm should be detected [13], but more open structures will require much more precise measurement [9].
- Simultaneous multi-point measurements to provide reliable information on the spatial distribution of gas concentrations.
- Adequate time resolution to detect changes determined by varying outdoor conditions.

From the livestock producer's point of view, it would be advisable to install a set of CO_2_ sensors at different points in a building. This would allow an in-depth study of CO_2_ concentration at these points and would monitor the airflow effect. Of course, to make this a viable approach, the measurement system must have a competitive price. Our proposal includes a wireless sensor network consisting of a set of CO_2_ sensors placed throughout the building. In order to select the most appropriate CO_2_ sensor for this work, an in-depth analysis of commercial sensors must be carried out. The sensor will then be connected to the best current technology to store and communicate the results: a wireless sensor network (WSN) architecture.

The huge advances in technologies such as very large scale integrated microelectromechanical systems and wireless communications have contributed to the widespread use of distributed sensor systems [18]. The miniaturization of computing and sensing technologies has enabled the development of tiny, low-powered, inexpensive sensors, actuators, and controllers. Embedded computing systems (*i.e.*, systems that typically interact closely with the physical world and are designed to perform only a limited number of dedicated functions) continue to find applications in an increasing number of areas. While defense and aerospace systems still dominate the market, there is an increasing focus on systems that monitor and protect civil infrastructures such as bridges and tunnels, the national power grid and pipelines. Networks of hundreds of sensor nodes are already being used to monitor large geographic areas for modeling and forecasting environmental pollution and flooding, using vibration sensors to collect structural health information on bridges, and controlling usage of water, fertilizers, and pesticides to improve crop health and quantity.

In this paper, we deal with two particular objectives. First, the selection of an appropriate CO_2_ sensor to be used in wireless sensor networks, and sly, to evaluate this sensor under laboratory conditions in order to assess its potential application to practical aspects of livestock farming, such as ventilation control, welfare assessment or environmental applications. The paper is structured as follows: Section 2 describes the selection process of the CO_2_ sensor and the wireless CO_2_ sensor network is presented in Section 3, including the experimental set-up for evaluating the sensor performance. Section 4 includes the results and discussion and, finally, our conclusions are presented in Section 5.

## 2. Choice of CO_2_ Sensor

The type of installation in which the sensors will be deployed requires the measurement of concentrations of CO_2_ in air for a range between 0 and 5000 ppm with an accuracy of ±100 ppm. The choice of sensor is critical, since our experimental set-up requires a wireless network of CO_2_ sensors. This imposes the following restrictions:

- Energy-constrained wireless sensor nodes. Both the CO2 sensors and wireless nodes must have low energy requirements. The set-up can vary from one recording per min every week to one every 10 min for five months, so that the battery system must be sized to last for a whole campaign.
- Small sensor size for embedding in the physical node.
- No recalibration while the experiment is running. Error specifications must be limited and known for the full experiment, which can mean as long as five months of known measurement behavior. Measurement accuracy must be as high as possible without the need for recalibration during the entire experiment [19].
- Long sensor life. Some CO2 sensors have a limited life due to chemical contamination. This degradation influences measurement, implying extra maintenance.
- Low-cost. To obtain simultaneous multi-point measurements a considerable number of nodes are needed, so sensor price is important.
- Accurate sensors. Known accuracy is necessary to detect differences in concentration.

Taking the above restrictions into consideration, the choice of the right sensors was based on an analysis of different CO_2_ measurement techniques. Technologies for measuring CO_2_ in the air can be grouped into two categories: electrochemical and spectral absorption.

Commercially available electrochemical sensors were ruled out, as they have a limited life and high energy requirements due to the need for preheating and stabilizing before use.

Spectral absorption-based non-dispersive infrared (NDIR) sensors seemed the most suitable for the scenarios of the study. NDIR sensors are widely employed in atmospheric CO_2_ concentration measurements since they are considered stable, have excellent durability and are robust against interference from other air components.

Low-cost NDIR [19] sensors are now widely available, however their specifications and long-term characteristics are in general vague or have not been authenticated.

Bearing in mind the requirements of our study, the market offer was analyzed and the specifications compared. Table 1 summarizes a selection of models that apparently suited our requirements.

Since energy consumption is an important restriction, we estimated the total amount of energy required for different configurations. It should be noted that a measurement requires initial preheat and stable sensor readings, which must be allowed for in the calculations.

Table 2 summarizes the results for different reading periods with a 1.5 volt 3000 mAh battery. Some modules need a DC-DC converter. The example in the table is for an output voltage of 5 V and efficiency of 80%. It should be remembered that in general lower voltages provide better energy efficiency.

**Figure 1.**
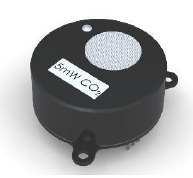
View of the SST Sensing CO2S-A sensor.

From the battery life point of view, the E2V IR11BD sensor seems the best option, but has serious drawbacks, such as the lack of an instrumentation amplifier (requiring its implementation in the node), so this part of the energy requirements is not evaluated. Also, the lack of double channel output means it needs continuous recalibrations. Based on considerations of energy, specifications and final sensor price, the CO2S-A model (SST Sensing Ltd., Coatbridge, UK) was chosen for the wireless node system. A view of this sensor is given in Figure 1.

## 3. Wireless CO_2_ Sensors Network

A wireless sensor network (WSN) consists of spatially distributed autonomous sensors to monitor physical or environmental conditions, such as temperature, sound, pressure, CO_2_, *etc.* and to pass the data acquired to a central personal computer (PC). As current networks are bi-directional, this means sensor activity can be controlled. Wireless sensor networks are now used in many industrial and consumer applications, such as industrial process monitoring, machine health monitoring, and so on.

The WSN is built of “nodes”—which can vary in number from a few to several hundred or even thousands, in which each node is connected to one or several sensors. Each sensor network node is typically divided into several parts: a radio transceiver with an internal antenna or connection to an external antenna, a microcontroller, an electronic circuit for interfacing with the sensors and an energy source, usually a battery or an embedded form of energy harvesting. A sensor node can vary in size from that of a shoebox down to the size of a grain of dust, although functioning “motes” of genuine microscopic dimensions have yet to be created. The cost of sensor nodes is similarly variable, ranging from a few to hundreds of euros, depending on the complexity of the individual sensor nodes. Size and cost constraints on sensor nodes result in corresponding constraints on resources such as energy, memory, computational speed and communications bandwidth. The topology of the WSNs can vary from a simple star network to an advanced multi-hop wireless mesh network. The propagation technique between the hops of the network can be routing or flooding [18,20].

**Figure 2.**
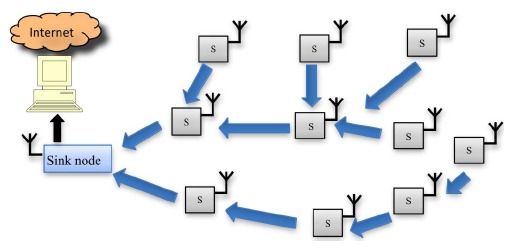
Wireless sensor network (WSN) scheme.

Figure 2 shows a typical scheme of a wireless sensor network. A set of nodes (S) transmits by a wireless protocol the information obtained through the network to the sink node. As can be seen in the Figure, some sensor nodes can act as repeaters, which allows low power transmit modes to be used to extend battery life. The sink node is connected by Universal Serial Bus (USB) or Ethernet to a personal computer to store the information received. Distance between sink nodes and nodes could be (using appropriate antennas and wireless technology) hundreds of meters.

### 3.1. System Architecture

**Figure 3.**
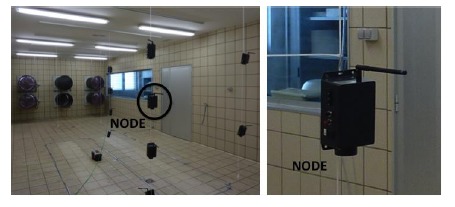
System deployment in a chamber.

To demonstrate the feasibility of using a wireless sensor network to sense CO_2_ levels, 12 wireless nodes were implemented and installed in a controlled chamber (Figure 3).

The chamber is equipped with a set of fans that can reproduce air currents. As can be seen in Figure 3, the nodes were installed in the form of a matrix to measure the CO_2_ at different heights.

### 3.2. Node Characteristics

**Figure 4.**
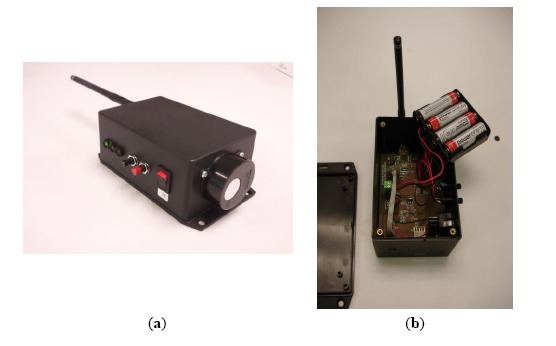
CO_2_ Node photographs: (**a**) External view; and (**b**) Internals.

Figure 4 shows two views of the wireless CO_2_ node, which must be installed in a vertical arrangement to ensure that the CO_2_ sensor is below the node box. It allows airflow through the sensor and is protected from dust.

Figure 4a also shows three Light Emission Diodes (LED), two pushbuttons and the power on/off button. LEDs and pushbuttons are used basically to program the time between measures (sample and transmit frequency to the sink node). This time can be configured by pressing one of the buttons and selecting one of the following values: 15, 30, 60, 120, 300, 600, 1200 and 3600 s.

Figure 4b shows the node printed circuit board and the battery pack that ensures a measurement campaign of several weeks. Battery levels are monitored and sent to the sink node to warn users if a battery replacement is necessary.

The node is based on the CC1110F32 microcontroller (Texas Instruments, Dallas, TX, USA) [21]. The CC1110F32, is a true low-power sub-1 GHz system-on-chip designed for low power wireless applications. It combines the excellent performance of the state-of-the-art RF transceiver CC1101 with an industry-standard enhanced 8051 MCU, up to 32 KB of in-system programmable flash memory and up to 4 KB of Random Access Memory (RAM), and many other powerful features. The radio frequency range can be chosen from: 300–348 MHz, 391–464 MHz and 782–928 MHz. The European Industrial, Scientific and Medical (ISM) band 868 MHz frequency and 500 kbps were chosen for the present implementation. This ISM band allows longer ranges than typical systems using 2.4 GHz (up to almost 3 times higher for the same transmit power and sensitivity reception). Additionally, its low power demands (16 mA for transmission at 10 mW and 18 mA for reception) make it suitable for battery-powered systems. In our tests, ranges up to 290 m with an omnidirectional antenna were achieved, which was in excess of the requirements in livestock buildings.

The Sensirion SHT11 [22] digital humidity and temperature sensor was included to analyze the influence of temperature and humidity on CO_2_ measuring. This is the all-round version of the reflow solderable humidity sensor series that combines decent accuracy with a competitive price and is fully calibrated. The information transmitted to the sink node was as follows:

- CO_2_ concentration
- Ambient temperature
- Relative humidity
- Battery level

For the first three items, the node software averaged the last ten measures before sending the result to the sink node.

### 3.3. Sink Node

**Figure 5.**
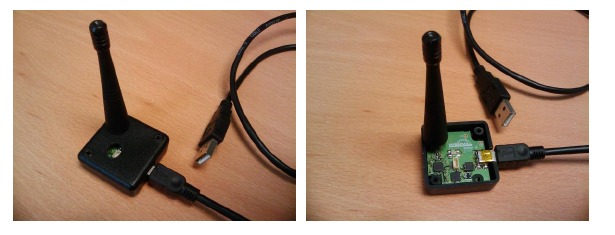
Views of the sink node.

The sink node (see Figure 5) receives all the information transmitted by the set of nodes and communicates this data by means of a USB connection to a personal computer that stores all the information received in a Comma Separated Values (CSV) format file that can be processed by many standard PC applications (database or spreadsheet software).

The sink node hardware is the same as that of the other nodes, without the CO_2_ and temperature and humidity sensors. As it is connected by a USB port to the PC it does not need batteries. Although a USB connection was chosen for this system, other alternatives such as Ethernet connection or General Packet Radio Services (GPRS) can be implemented to send information to the PC.

**Figure 6.**
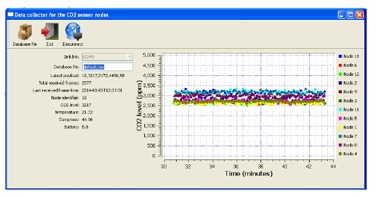
Data collection software interface.

Figure 6 shows the user-friendly application used to store the acquired information in a CSV database.

### 3.4. Assessment of Sensor Performance

**Figure 7.**
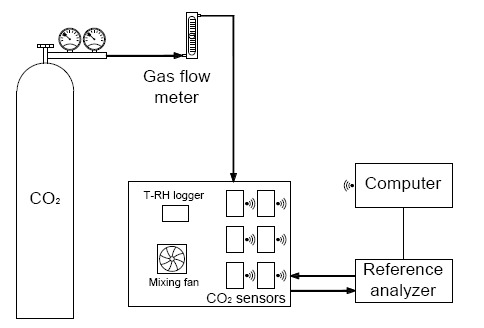
Experimental setup including closed chamber, gas source and reference analyzer.

The next step consisted of the calibration and evaluation of sensors and a number of different tests under laboratory conditions to evaluate the performance of the wireless sensor network. The sensors were calibrated in a closed chamber measuring 50 cm long × 30 cm wide × 30 cm high lined with poly methylmethacrylate tiles under laboratory conditions. The chamber was equipped with a fan to homogenize the air inside it (Figure 7). Pure CO_2_ was injected into the chamber at a constant rate of 1 L per min until the target concentration was achieved. To reduce concentrations inside the chamber, fresh air was forced in at a constant rate of 2 L/min. The concentrations could be maintained inside the chamber when both flows were stopped. A photoacoustic gas analyzer (Innova 1412, LumaSense Technologies, Ballerup, Denmark) was used as a reference for CO_2_ concentration. This device has a precision of 1.5 ppm, which is considerably higher than the expected precision of the CO_2_ sensor network. Temperature and relative humidity were also measured by means of a data logger (HOBO U12-013, OnsetComp, Bourne, MA, USA). All these measurements were taken at a 1-min frequency, while the CO_2_ WSN registered information every 15 s. The different tests performed are described below.

Preliminary tests were conducted during sensor selection to evaluate linearity and potential interference under normal livestock-rearing conditions. Linearity was tested within the range from 500 to 5000 ppm by gradually varying gas concentration. Cross effects of temperature (16 °C, 24 °C and 32 °C) and relative humidity (15%, 40% and 60%) were assessed. As CH_4_ is normally present in livestock buildings as a result of fermentation processes it could potentially affect sensor readings as it also absorbs infrared radiation. Potential cross interference was evaluated by introducing this gas into the chamber from a calibration bottle (60% CH_4_–40% CO_2_) until 100 ppm were reached. The effect of the sensor node battery status was also monitored at values of 6.3 V (full battery), 5.7 V, 5.3 V and 4.9 V. An analysis of variance (ANOVA) was conducted to evaluate significant differences which could indicate cross interference by these factors.

As the preliminary tests showed that the sensor had a highly linear response to CO_2_ concentrations within the range from 500 to 5000 ppm, a three point calibration was conducted at approximately levels of 600, 2400 and 4000 ppm. The calibration consisted of placing all the sensors inside the chamber and injecting CO_2_ as described above until a constant value was reached by both the reference analyzer and the sensors under study. Once equilibrium was reached, each level was maintained for 30 min. All the tests were conducted at 24 °C ambient temperature and 50% relative humidity. The full procedure was repeated four times.

The calibration curve for each sensor was obtained by simple regression using the measurements for each sensor as a dependent variable and the corresponding measurements of the reference analyzer as independent variable. Homogeneity between the sensors was tested using a multiple regression which included sensor influence on intercept and slope as dummy variables, according to the following model:

(1) $${\text{CO}}_{2\text{Sensor}}=\left(\beta_{0}+\beta_{i}\right)+\left(\beta_{1}+\beta_{j}\right)\times {\text{CO}}_{2\text{ref}}+ɛ$$

where CO_2 sensor_ is the sensor reading, CO_2 ref_ is the reading of the reference analyzer (ppm), β_0_ and β_1_ are the intercept and slope of the calibration of a specific sensor, and β*_i_* and β*_j_* are the differences in intercept and slope of a comparison sensor. Significant values of β*_i_* and/or β*_j_* thus represent significant differences between sensors.

From this test sensor precision was also obtained at the different CO_2_ levels tested for calibration (600, 2400 and 4000 ppm). Following ISO 3534-2:2006 [23], precision was defined as the standard deviation of concentrations given by the sensor (after applying the calibration curve). According to this standard, this represents repeatability, *i.e.*, the precision under observation conditions in which independent results are obtained by the same method on identical measurement items in the same test by the same operator and equipment within short intervals of time.

The time response of the sensor to a sharp decrease in ambient CO_2_ concentration was also studied. Sensors were maintained for 30 min inside the chamber at a constant CO_2_ concentration of about 5000 ppm. After this period, they were removed from the chamber and exposed to fresh air (CO_2_ concentration about 400 ppm) in still air (air velocity = 0 m/s), after which sensor readings were taken for another 30 min. Temperature and relative humidity did not show relevant changes (less than 0.2 °C and less than 1%, respectively) between both sensor positions (inside and outside the chamber).This procedure was repeated 9 times. According to the preliminary tests, 30 min was enough for the sensor to stabilize. During this changeover period, the sensor signal followed a decay curve according to the following general equation:

(2) $$C_{t}=C_{\text{fin}}+\left(C_{\text{ini}}-C_{\text{fin}}\right)\times e^{-\text{kt}}$$

where *C_t_* is the CO_2_ concentration (ppm) at a certain time “*t*” (min) from the beginning of the test, *C*_ini_ and *C*_fin_ are the initial and final CO_2_ concentrations, respectively, and *k* is the model constant (min^−1^). To obtain the model constant Equation (2) was linearized by logarithms and a simple linear regression model was obtained:

(3) $$\text{Ln}\left(\frac{C_{t}-C_{\text{fin}}}{C_{\text{ini}}-C_{\text{fin}}}\right)=k\times t$$

Once *k* had been obtained, the average times needed to achieve a 90% and 95% change in readings were determined.

## 4. Results and Discussion

**Figure 8.**
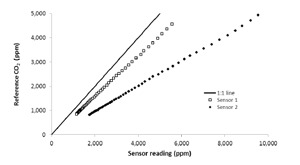
Sensor response to varying CO_2_ concentrations (range from 1000 to 5000 ppm).

Preliminary tests on two sensors demonstrated that the sensor was highly linear within the range from 500 to 5000 ppm, which includes most practical situations in livestock operations (Figure 8). In all the tests a very good linear response (*R*^2^ > 0.99) was obtained from the sensor. However, these preliminary tests also revealed very relevant differences among sensor performance (see different slopes for sensors 1 and 2 in Figure 8).

Regarding potential cross effects, temperature, relative humidity and battery status did not have any effect on CO_2_ readings at the studied levels (*p* > 0.05). However, exposure to 100 ppm of CH_4_ caused a sensor underestimation of about 10% in CO_2_ readings. This gas may accumulate in livestock buildings, particularly in ruminant houses and manure storage facilities. Although in poultry production CH_4_ concentrations are typically lower than 10 ppm [14,16], in dairy cow barns it may be as high as 40 ppm [24]. Specific research should be conducted to determine with precision how exposure to different levels of CH_4_ affects NDIR CO_2_ sensor readings under practical farm conditions.

Calibration tests of all 12 sensors indicated varying responses of individual sensors to the same CO_2_ concentrations (Table 3). Significant differences were noted between all the sensors, which suggests the need for individual calibration. On the other hand, temperature and relative humidity readings showed only non-significant differences. The temperature differences were always less than 0.4 °C, whereas for relative humidity the differences consistently lower than 1.5%.

Sensor calibration parameters are indicated in Table 4. As suggested by the results in Table 3, calibration curves were statistically different between sensors (*p* < 0.05), which meant a specific calibration equation was required for each sensor. As in the preliminary results, all the calibrations showed a very good linear relationship (*R*^2^ was consistently over 0.99). The standard error of estimation (SEE) obtained by the regression model is an estimation of sensor precision and represents the deviations of sensor readings in comparison with reference values. As indicated above, this value represents sensor repeatability. Sensor precision ranged from 58.2 ppm (Sensor #1) to 117 ppm (Sensor #11), most of the sensors being within the 80 to 110 ppm range.

One of the most positive sensor characteristics is its very high linearity, which allows two- or three-point calibration. However, the response was found to vary significantly from one sensor to another, so that individual calibrations were required. Sensor precision measured under laboratory conditions was about 2 times the catalog value (50 ppm).

Field experiments should be conducted to determine whether this precision is maintained under non-controlled conditions. This degree of precision may be enough for welfare assessment, when 3000 to 5000 ppm are established as threshold values. This sensor may also be sufficiently reliable to implement climate control systems using CO_2_ as a ventilation criterion. In such systems, ventilation comes into operation when CO_2_ exceeds a specific value (e.g., 3000 ppm in broiler houses, according to European Council Directive 2007/43/EC) [11].

However, these sensors could also be used to conduct CO_2_ balances to indirectly estimate ventilation flows under certain conditions. As indicated by Ouwerkerk and Pedersen in [13], the CO_2_ measurement system should be accurate enough to detect differences of at least 150 ppm between air outlets and inlets. In practice, CO_2_ balances are easier to conduct when the difference in concentration is higher, as in enclosed animal buildings, which normally have well defined air inlets and outlets. However, the great potential of CO_2_ balances may be associated with relatively open buildings, in which CO_2_ differences may be lower than 150 ppm and which may even be subject to incomplete mixing of fresh and polluted air. For example, [16,17] reported buildings with average CO_2_ concentrations lower than 500 ppm in pig and poultry houses, whereas [25] reported an average concentration of 553 ppm in a dairy cow house, where indoor CO_2_ concentrations as low as 313 ppm (similar to outdoor concentration) were measured. Similar results were reported by [24]. Unfortunately, this sensor may not be accurate enough in such cases.

**Figure 9.**
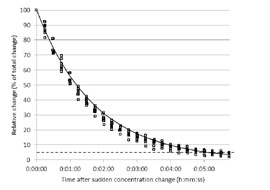
Sensor time response, expressed in relative terms: 100% represents the initial concentration and 0% the final concentration.

The sensor response time is shown in Figure 9, which represents the decrease in concentration expressed in relative terms, from 100% (initial concentration) to 0% (final or equilibrium concentration) when concentrations suddenly change from high to low. The time necessary to achieve a specific change of concentration can be obtained from this curve. According to Equation (3), a decay constant *k* = 0.582 min^−1^ was obtained. Following this model, it was determined that a 90% change in concentration is obtained in 3 min and 57 s, whereas a 95% change is obtained in 5 min and 8 s. According to the curves shown in Figure 9, it can be concluded that this behavior was constant for all the sensors.

A similar response time to that obtained in the present study was also obtained in [19], whose authors tested five different NDIR CO_2_ sensors and obtained 90% response times varying from less than 1 min to 3 min and 10 s, which is considerably lower than the time response of the SST CO2S-A sensor used in this study. The sensor response obtained by us is adequate for questions of animal welfare, considering that indoor CO_2_ concentrations are relatively constant in most livestock buildings. It can also be considered acceptable for characterizing the indoor climate and measuring emissions in most mechanically-ventilated livestock buildings, in which air fluxes tend to be stable. However, this response is too slow to determine CO_2_ fluxes in naturally ventilated buildings. These buildings are characterized by variable and unpredictable changes in flow directions, in such a way that air inlets and outlets may change in short time intervals as a consequence of turbulence and changes in outdoor conditions. For this reason, the boundary conditions of these buildings normally involve sudden changes in CO_2_ concentrations, from relatively high (when an opening is acting as an air outlet) to low, background CO_2_ concentrations (when air is entering).

As reported in [19], the output of NDIR sensors is affected by the length of time they are in use. They found a linear relationship in sensor response as a function of time when used for less than one year, which may be considered correct sensor output. Although this effect was not analyzed in our study, preliminary information suggests that this effect may also occur with the sensors we used. In this sense, some significant differences can be found in calibration curves separated in time more than one week. Therefore, must be conducted to evaluate the long-term performance of these sensors. Frequent sensor calibrations would thus be advisable before use. Response time could also be affected by the length of time in use. Although [19] did not find any clear relationship between these two variables, it must be considered that sensor response is affected by gas diffusion through a membrane to the sensor chamber, so that the factors affecting membrane permeability (e.g., dust accumulation) may slow down the sensor response. According to [26,27], in these sensors there is a potential cross interference with dust due to light scattering and therefore sensors are placed in closed or diffusion cells protected by dust filters. For this reason, dust accumulation in these filters may reduce diffusion to the measurement cell, thus affecting response time of these NDIR sensors

## 5. Conclusions

This paper describes a study on the use of a low cost CO_2_ sensor implemented in a wireless sensor network system that showed an excellent throughput during measurement campaigns. This system combines moderate cost with low energy requirements and can be powered by standard batteries, which simplifies its installation. A set of nodes can be placed at different points throughout a livestock building to acquire readings. The nodes are also designed to make it possible to include other CO_2_ sensors or additional sensors to detect the presence of other gases (*i.e.*, methane).

The CO_2_ sensor selected may be appropriate for animal welfare and environmental applications in livestock production, particularly in animal houses which are relatively open to the atmosphere. The sensors were seen to respond linearly to changes in CO_2_ concentrations. However, the differences between individual sensors and a potential time drift make it advisable to calibrate before a measurement campaign. The sensor time response (about 5 min) makes it appropriate for most applications, except those requiring a fast response. For this reason these sensors may not be appropriate to monitor slight, sudden changes occurring in open, naturally ventilated buildings.

## Figures and Tables

**Table 1.** List of non-dispersive infrared (NDIR) CO_2_ sensors considered.

Manufacturer	Hanwei	SST Sensing	Alphasense	Dynament	E2V	Senseair
Model	MH-Z12	CO2S-A	IRC-A1	TDS0037	IR11BD	K30
Measurement range (ppm)	0–5,000	0–5,000	0–5,000	variable	variable	0–10,000
Accuracy	±50 ppm	±50ppm ±3% measure	1%	2%	±100 ppm	±30ppm ±5% measure
Response time (s)	<30 + 180 preheat	30 to 180	<40	< 30 + 60 preheat	< 20 + 30 to 1800 preheat	20
Operating voltage (V)	4–6	3.25–5	2–5	3–5	3–15	4.5–14
Size (mm) (Height × height × depth /Ø Diameter × height)	60 × 42 × 15	Ø 60 × 20	Ø 20 × 17	Ø 20 × 17	Ø 14 × 19	51 × 51 × 41
Approximate price (Euros)	120	80	75	100	150	70

**Table 2.** Estimated life in days for 1.5 V, 3000 mAh battery, DC-DC 80% efficiency and 5 V sensors.

Measure interval (min)	Model					

	Hanwei MH-Z12	SST Sensing CO2S-A	Alphasense IRC-A1	Dynament TDS0037	E2V IR11BD	Senseair K30
Cont.	1.2	3.6	1.2	0.9	2	1.8
5	1.7	7.2	5.1	3.0	15.0	6.0
10	3.4	14.4	10.3	6.0	30.0	12.0
15	5.1	21.6	15.4	9.0	45.0	18.0
20	6.9	28.8	20.6	12.0	60.0	24.0
25	8.6	36.0	25.7	15.0	75.0	30.0
30	10.3	43.2	30.9	18.0	90.0	36.0
120	41.1	172.8	123.4	72.0	360.0	144.0
360	123.4	518.4	370.3	216.0	1080.0	432.0

**Table 3.** Sensor readings (average ± standard deviation) at three different CO_2_ levels. Concentrations obtained by the reference analyzer are also shown. Units are expressed in ppm.

CO_2_ level	600	2400	4000
Reference	607.9 ± 2.0	2386.5 ± 2.1	4039.4 ± 4.3
Sensor #1	2328.0 ± 2.1	5823.5 ± 4.0	9153.7 ± 6.9
Sensor #2	841.6 ± 2.1	3013.3 ± 4.0	5073.8 ± 6.3
Sensor #3	774.1 ± 2.1	2843.2 ± 4.0	4885.5 ± 6.3
Sensor #4	1976.8 ± 2.1	5133.0 ± 4.0	8280.0 ± 6.3
Sensor #5	943.2 ± 2.1	3150.3 ± 4.0	5339.7 ± 6.3
Sensor #6	743.1 ± 2.1	2751.7 ± 4.0	4762.0 ± 6.3
Sensor #7	866.6 ± 2.1	2992.8 ± 4.0	5111.2 ± 6.3
Sensor #8	828.0 ± 2.1	2951.6 ± 4.0	5079.9 ± 6.3
Sensor #9	978.7 ± 2.1	3164.2 ± 4.0	5274.7 ± 6.3
Sensor #10	1219.9 ± 2.1	3547.7 ± 4.0	5759.0 ± 6.3
Sensor #11	1333.6 ± 2.1	3779.1 ± 4.0	6132.3 ± 6.3
Sensor #12	822.0 ± 2.1	2921.5 ± 4.0	4993.7 ± 6.3

**Table 4.** Calibration parameters of individual sensors. The calibration model is CO_2_ corrected = β_0_ + β_1_ × CO_2 sensor_ + ε. Standard errors of coefficients are indicated in parenthesis. The determination coefficient (*R*^2^) and the standard error of estimation (SEE) are also indicated.

CO_2_ level	β_0_	β_1_	*R*^2^	SEE (ppm)
Sensor #1	−537.4 (3.6)	0.498 (0.0006)	0.998	58.2
Sensor #2	−60.46 (3.9)	0.808 (0.001)	0.997	74.8
Sensor #3	−6.6 (4.7)	0.829 (0.001)	0.996	91.5
Sensor #4	−438.3 (4.6)	0.543 (0.001)	0.997	80.3
Sensor #5	−88.7 (5.7)	0.774 (0.002)	0.994	109.0
Sensor #6	7.2 (4.6)	0.849 (0.001)	0.996	91.0
Sensor #7	−57.2 (4.5)	0.803 (0.001)	0.996	87.5
Sensor #8	−24.5 (4.3)	0.802 (0.001)	0.997	83.3
Sensor #9	−144.9 (5.5)	0.793 (0.001)	0.995	102.3
Sensor #10	−283.9 (6.1)	0.749 (0.002)	0.994	110.5
Sensor #11	−309.0 (6.6)	0.708 (0.002)	0.993	117.1
Sensor #12	−37.0 (4.5)	0.818 (0.001)	0.996	86.6
